# Modeling and Simulation of the Simultaneous Absorption/Stripping of CO_2_ with Potassium Glycinate Solution in Membrane Contactor

**DOI:** 10.3390/membranes10040072

**Published:** 2020-04-16

**Authors:** Nayef Ghasem

**Affiliations:** Department of Chemical and Petroleum Eng., UAE University, Al-Ain PO Box 15551, UAE; nayef@uaeu.ac.ae

**Keywords:** membrane contactor, CO_2_ absorption/stripping, potassium glycinate, numerical simulation

## Abstract

Global warming is an environmental problem caused mainly by one of the most serious greenhouse gas, CO_2_ emissions. Subsequently, the capture of CO_2_ from flue gas and natural gas is essential. Aqueous potassium glycinate (PG) is a promising novelty solvent used in the CO_2_ capture compared to traditional solvents; simultaneous solvent regeneration is associated with the absorption step. In present work, a 2D mathematical model where radial and axial diffusion are considered is developed for the simultaneous absorption/stripping process. The model describes the CO_2_/PG absorption/stripping process in a solvent–gas membrane absorption process. Regeneration data of rich potassium glycinate solvent using a varied range of acid gas loading (mol CO_2_ per mol PG) were used to predict the reversible reaction rate constant. A comparison of simulation results and experimental data validated the accuracy of the model predictions. The stripping reaction rate constant of rich potassium glycinate was determined experimentally and found to be a function of temperature and PG concentration. Model predictions were in good agreement with the experimental data. The results reveal that the percent removal of CO_2_ is directly proportional to CO_2_ loading and solvent stripping temperature.

## 1. Introduction

Carbon dioxide (CO_2_) is the main contributor to global warming, which is a worldwide concern. Pre- and post-combustion capture of CO_2_ is the main solution to avoid this problem. The prominent technology nowadays is the absorption of CO_2_ in amine solution, such as monoethanolamine (MEA) and diethanolamine (DEA) that takes place in a reversible chemical reaction. Despite the success of these chemicals in absorbing CO_2_ from natural gas and flue gas, they suffer from certain drawbacks such as the regeneration of the alkanolamine solutions, which require high-energy consumption, solvent evaporation and degradation losses, and corrosion to pipes and equipment [1,2,3]. An alternative solvent of CO_2_ capture is the amino acid salts such as potassium glycinate (PG) [4]. The PG aqueous solvents have high reactivity toward CO_2_ and less regeneration energy consumption [5], the aqueous PG solvent has a comparable functional group as alkanolamine. The amino acid salts overcome the drawbacks of the alkanolamine solution in terms of low volatility because of their ionic structure, high surface tension, and low opportunity to degradation due to their resistance to oxidative degradation [6]. The reaction mechanism and reaction rate constants are essential for the simulation, evaluation, and design of the absorption/stripping process of CO_2_ in potassium glycinate (PG) in conventional absorption towers and solvent–gas membrane separation processes. Several researchers have studied the kinetics of these amino acid salts (AAS) [7,8,9,10,11,12,13]. Absorbent based on potassium glycinate showed higher reactivity than sodium based absorbent toward CO_2_ [14]. The kinetic data of the absorption of CO_2_ in several AAS are summarized elsewhere [8]. 

The traditional packed bed and plate column and cryogenic are the most commonly used techniques for the removal of CO_2_ gas from gaseous mixtures in alkanolamine solutions [5,15,16]. In spite of the success of these units, they suffered from certain weaknesses such as flooding, channeling, foaming, and high operating cost [17]. The alternative emerged technique is the solvent–gas membrane interaction system that can compete with the traditional solvent system; the membrane has a small structure size, liquid absorbent and gas stream flow in separate channels, is easy to scale up, and a high surface area per unit volume [2,17,18,19]. Many researchers recommend a hollow fiber membrane contacting system for the absorption and stripping of CO_2_ from rich solvent because of its noticeable advantages compared to conventional processes [13,16,20,21,22,23]. The effective absorption of CO_2_ in lean solvent and the desorption process of CO_2_ from rich liquid solvent (regeneration process) in hollow fiber membrane contactor depends preferably on the high membrane porosity and less on membrane moistening as wetting depends mainly on the reactivity of membrane material with solvent being used. Accordingly, the membrane material is preferred to be from hydrophobic polymeric material such as polyvinyl fluoride (PVDF) [1,22,24,25]. On the other hand, the absorbent liquid is designated to have high surface tension to prevent the penetration of liquid into the pores of the membrane [26]. Amino acid solvents have high surface tension properties such as glycinate family, potassium glycinate (PG), and sodium glycinate [23,27,28,29,30]. Membrane fabricated from polyetherimide (PEI), polysulfone (PS), and polytetrafluoroethylene (PTFE) have acceptable CO_2_ absorption and stripping efficiency and have high liquid entry pressure and, hence, were used for absorption and regeneration of carbon dioxide from flue and natural gas by several researchers [22,25,31,32]. 

Most of the literature work has focused on modeling and simulation of CO_2_ absorption in membrane contactors; less attention has been given to CO_2_ stripping from rich solvents in membrane contactors [16,33,34,35,36], and almost none were established for simultaneous modeling and simulation of the absorption/stripping process in the solvent–gas membrane separation process. Accordingly, the purpose of this work is the modeling and simulation of the simultaneous absorption/stripping of CO_2_ in lean and rich PG aqueous solution in membrane contactor, respectively. The validated model was used to study the effect of gas and solvent flow rates, solvent temperature, and CO_2_ loading in rich PG on membrane separation efficiency. The simulation results were compared with experimental data.

## 2. Model Development

**Figure 1:**
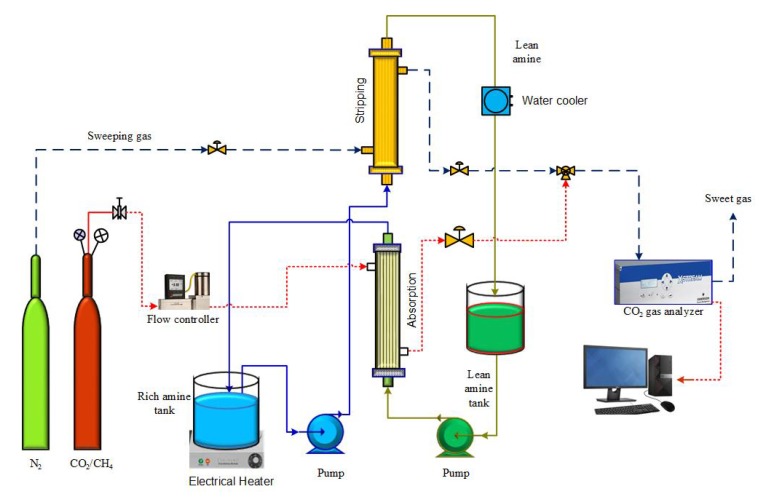
Schematic of the absorption/stripping process using a gas–solvent membrane system.

The model equations that describe the absorption/stripping of CO_2_ in aqueous PG through a gas–solvent membrane contactor were developed. Table 1 outlines the dimensions of the hollow fiber membrane used in the experimental and model development. Figure 1 shows the experimental setup for the absorption of CO_2_ in potassium glycinate solvent and stripping of rich solvent using solvent-gas membrane contacting modules for both absorption and stripping. The feed is 10% CO_2_/90% CH_4_ gas mixture flows into the membrane module shell side at variable gas inlet flow rate adjusted by Alicat mass flow controllers (Alicat Scientific, Tucson, AZ, USA). The solvent was 0.5 M aqueous potassium glycinate. The inlet solvent flow rate to absorber is monitored by a peristaltic pump (Masterflex L/S). The solvent (0.5 MPG) is supplied to the membrane lumen side at variable feed rates. The effluent of the absorber is heated to temperature range 20–80 °C. The heated stream is supplied to the tube side of the stripper (membrane module). The sweeping gas is nitrogen; the exit gas concentrate is measured using gas chromatography (Shimadzu, Kyoto, Japan) and a CO_2_ gas analyzer (CAI, 600 Series, Orange, CA, USA). 

**Figure 2:**
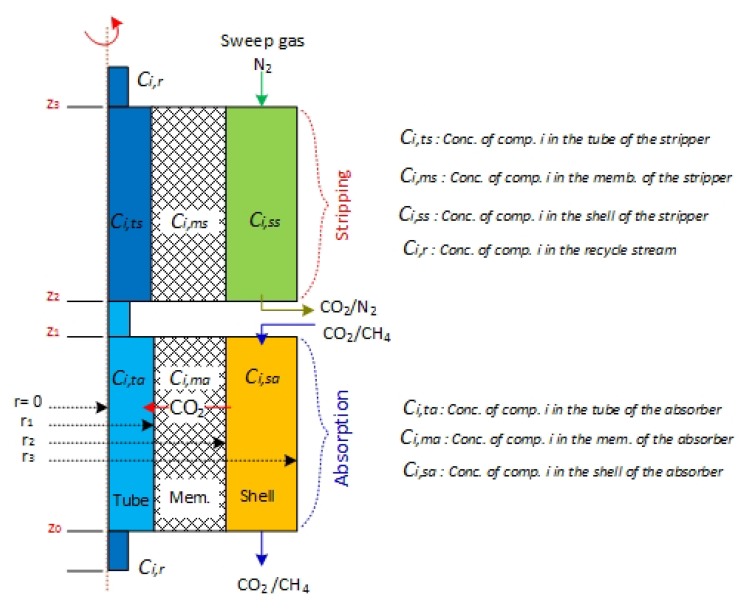
Schematic of the membrane module used in modeling the absorption/stripping system.

The developed 2D axisymmetric, transient mathematical model describes the absorption/stripping process in the solvent–gas hollow fiber membrane contacting process (Figure 2), considering a cylindrical coordinate, isothermal, and nonwetting mode of operation assumptions. While absorption operates at room temperature, stripping operates at variable solvent feed temperatures (25–80 °C). The model considers the ideal gas, incompressible liquid, and Newtonian fluid assumptions. Henry’s law measures the solubility of CO_2_ in the solvent at the solvent–gas interface. The CO_2_ and PG in the liquid phase is transported in the tube side by both diffusion and convection. CO_2_ diffuses across the membrane film by diffusion only. The following mass transport equations describe the absorption of the CO_2_ in lean PG and the stripping of CO_2_ from rich PG solvent.

In the model development, the subscripts in the material balance equations, ta, ma, sa, refers to the tube, membrane, and shell sides of the absorber, respectively, where ts, ms, ss, refers to the tube, membrane, and shell sides of the stripper, respectively. For example, $C_{CO_{2},ta}$ refers to the concentration of CO_2_ of the solvent present in the tube side of the absorber module. PG is the aqueous liquid solvent, $r_{i}$ is the forward reaction of component i, and $R_{i}$ is the reverse reaction rate of component i. 

### 2.1. Absorption

#### 2.1.1. Hollow Fiber Lumen

The mass balance equation for CO_2_ in PG rich solvent flowing in the tube side is described by Equation (1):

(1) $$\frac{\partial C_{CO_{2},ta}}{\partial t}=D_{CO_{2},t}\left[\frac{\partial^{2}C_{CO_{2,ta}}}{\partial r^{2}}+\frac{1}{r}\frac{\partial C_{CO_{2},ta}}{\partial r}+\frac{\partial^{2}C_{CO_{2},ta}}{\partial z^{2}}\right]+r_{CO_{2},ta}-v_{z,t}\frac{\partial C_{CO_{2},ta}}{\partial z}$$

The solvent circulation velocity ($v_{z,t}$) is described by the parabolic equation:

(2) $$v_{z,t}=\frac{2Q_{t}}{n\pi r_{1}^{2}}\left(1-{\left(\frac{r}{r_{1}}\right)}^{2}\right)$$

*Q_t_* is the solvent circulation volumetric rate, and n is the number of fibers.

The boundary settings:

(3) $$\mathrm{at}\;z=z_{0},\;C_{CO_{2,ta}}=C_{CO_{2,tr}}\;(\mathrm{concentration}\;\mathrm{of}\;{\mathrm{CO}}_{2}\;\mathrm{in}\;\mathrm{rich}\;\mathrm{PG}\;\mathrm{from}\;\mathrm{recycling})$$

(4) $$\mathrm{at}\;z=z_{1},\;\frac{\partial^{2}C_{CO_{2},ta}}{\partial z^{2}}=0\;(\mathrm{convective}\;\mathrm{flux})$$

(5) $$\mathrm{at}\;r=0,\;\frac{\partial C_{CO_{2,t}}\;}{\partial r}=0\;(\mathrm{axis}\;\mathrm{symmetry})$$

(6) $$\mathrm{at}\;r=r_{1},\;C_{CO_{2},ta}=m\;C_{CO_{2},ma}\;(\mathrm{solubility})$$

Since the PG contain amino groups similar to traditional amines, the reaction between CO_2_ and PG can be described by the zwitterion mechanism [21,37].

(7) $$CO_{2}+H_{2}N-CHR^{\prime }-COO^{-}K^{+}\left(PG\right)↔\;COO^{+}H_{2}N-CHR^{\prime }-COO^{-}K^{+}\left(Pg-CO_{2}\right)$$

The forward reaction rate is expressed as follows [36].

(8) $$r_{CO_{2}}=-2.42\times 10^{16}\exp\left(\frac{-8544}{T}\right)\exp\left(0.44C_{Pg}\right)C_{Pg}C_{CO_{2}}$$

where $C_{pg}$ and $C_{CO_{2}}$ are the concentrations of PG and CO_2_; *T* (K) is the liquid temperature.

#### 2.1.2. Membrane Layer

The mass transfer of solute gas (CO_2_) in the membrane section bounded between $r_{1}$ and $r_{2}$ is expressed in Equation (9) by diffusion only [38]:

(9) $$\frac{\partial C_{CO_{2},ma}}{\partial t}=D_{CO_{2},m}\left[\frac{\partial^{2}C_{CO_{2,ma}}}{\partial r^{2}}+\frac{1}{r}\frac{\partial C_{CO_{2},ma}}{\partial r}+\frac{\partial^{2}C_{CO_{2},ma}}{\partial z^{2}}\right]$$

Equation (10) describes the material balance for the CH_4_ in membrane layer

(10) $$\frac{\partial C_{CH_{4},ma}}{\partial t}=D_{CH_{4,m}}\left[\frac{\partial^{2}C_{CH_{4,ma}}}{\partial r^{2}}+\frac{1}{r}\frac{\partial C_{CH_{4,ma}}}{\partial r}+\frac{\partial^{2}C_{CH_{4,ma}}}{\partial z^{2}}\right]$$

Equations (11) to (14) are the boundary conditions of the membrane layer ($i:{\mathrm{CO}}_{2},{\;\mathrm{CH}}_{4}$)

(11) $$\mathrm{at}\;z=z_{0},\;\frac{\partial C_{i},ma\;}{\partial z}=0$$

(12) $$\mathrm{at}\;z=z_{1},\;\frac{\partial C_{i},ma\;}{\partial z}=0$$

(13) $$\mathrm{at}\;r=r_{1},\;D_{i,m}\frac{\partial C_{i},ma\;}{\partial r}=D_{i,t}\frac{\partial C_{i},ta\;}{\partial r}$$

(14) $$\mathrm{at}\;r=r_{2},\;C_{i,ma}=C_{i,sa}$$

#### 2.1.3. Shell of the Module

Equations (15) and (16) express the mass transfer of CO_2_ and CH_4_ gas in the shell side, respectively:

(15) $$\frac{\partial C_{CO_{2},sa}}{\partial t}=D_{CO_{2},s}\left[\frac{\partial^{2}C_{CO_{2,sa}}}{\partial r^{2}}+\frac{1}{r}\frac{\partial C_{CO_{2},sa}}{\partial r}+\frac{\partial^{2}C_{CO_{2},sa}}{\partial z^{2}}\right]-v_{z,s}\left(\frac{\partial C_{CO_{2},sa}}{\partial z}\right)$$

(16) $$\frac{\partial C_{CH_{4},sa}}{\partial t}=D_{CH_{4},s}\left[\frac{\partial^{2C}CH_{4},sa}{\partial r^{2}}+\frac{1}{r}\frac{\partial C_{CH_{4},sa}}{\partial r}+\frac{\partial^{2}C_{CH_{4},sa}}{\partial z^{2}}\right]-v_{z,s}\left(\frac{\partial C_{CH_{4},sa}}{\partial z}\right)$$

The velocity of gas in the shell side is estimated by [39]:

(17) $$v_{z,s}=v_{z,max}\left\{1-{\left(\frac{r_{2}}{r_{3}}\right)}^{2}\right\}\left\{\frac{{\left(\frac{r}{r_{3}}\right)}^{2}-{\left(\frac{r_{2}}{r_{3}}\right)}^{2}-2ln\left(\frac{r}{r_{2}}\right)\;}{3+{\left(\frac{r_{2}}{r_{3}}\right)}^{4}-4{\left(\frac{r_{2}}{r_{3}}\right)}^{2}+4\ln\left(\frac{r_{2}}{r_{3}}\right)}\right\}$$

The appropriate boundary conditions are as follows:

(18) $$z=z_{1},\;C_{i,sa}=C_{i,0}\;(\mathrm{inlet}\;{\mathrm{CO}}_{2}\;\mathrm{gas}\;\mathrm{concentration},\;8\;\mathrm{mole}/m^{3})$$

(19) $$z=z_{0},\;\frac{\partial^{2}C_{i},sa}{\partial z^{2}}=0\;(\mathrm{convective}\;\mathrm{flux})$$

(20) $$r=r_{2},\;D_{i,s}\;\frac{\partial C_{i},sa\;}{\partial r}=D_{i,ms}\;\frac{\partial C_{i},ma\;}{\partial r}\;(\mathrm{diffusive}\;\mathrm{flux})$$

(21) $$r=r_{3},\;\frac{\partial C_{i},sa\;}{\partial r}=0\;(\mathrm{symmetry})$$

The radius of the free surface ($r_{3}$), is expressed as follows:

(22) $$r_{3}=r_{2}{\left(\frac{1}{1-\varphi }\right)}^{0.5}$$

The module void fraction (φ):

(23) $$\varphi =\frac{R^{2}-n\;r_{2}^{2}}{R^{2}}$$

where R, $r_{2}$, n are the inner radius of the module, fiber outer radius, and the number of fibers, respectively. 

### 2.2. Stripping

#### 2.2.1. Hollow Fiber Lumen

The mass balance equation for CO_2_ in PG rich solvent in the tube side of the stripper membrane module is described by Equation (24):

(24) $$\frac{\partial C_{CO_{2},ts}}{\partial t}=D_{CO_{2},ts}\left[\frac{\partial^{2}C_{CO_{2,ts}}}{\partial r^{2}}+\frac{1}{r}\frac{\partial C_{CO_{2},ts}}{\partial r}+\frac{\partial^{2}C_{CO_{2},ts}}{\partial z^{2}}\right]-v_{z,t}\frac{\partial C_{CO_{2},ts}}{\partial z}+R_{CO_{2},ts}$$

where the velocity of liquid inside the hollow fibers ($v_{z,t}$) is described by the parabolic equation:

(25) $$v_{z,t}=\frac{2Q_{t}}{n\pi r_{1}^{2}}\left(1-{\left(\frac{r}{r_{1}}\right)}^{2}\right)$$

where *Q_t_* is the liquid solvent volumetric flow rate in the tube side, and n is the number of hollow fibers.

The appropriate set of boundary conditions are outlined as follows:

(26) $$\mathrm{at}\;z=z_{1},\;C_{CO_{2,ts}}=C_{CO_{2,ta}}\;(\mathrm{concentration}\;\mathrm{of}\;{\mathrm{CO}}_{2}\;\mathrm{in}\;\mathrm{rich}\;\mathrm{PG}\;\mathrm{exits}\;\mathrm{the}\;\mathrm{absorber})$$

(27) $$\mathrm{at}\;z=z_{2},\;\frac{\partial^{2}C_{CO_{2},ts}}{\partial z^{2}}=0\;(\mathrm{convective}\;\mathrm{flux})$$

(28) $$\mathrm{at}\;r=0,\;\frac{\partial C_{CO_{2,t}}\;}{\partial r}=0\;(\mathrm{axis}\;\mathrm{symmetry})$$

(29) $$\mathrm{at}\;r=r_{1},\;C_{CO_{2},ts}=m\;C_{CO_{2},ms}\;(\mathrm{solubility})$$

where *m* is the distribution coefficient ($m=8.314\times T/H_{CO_{2}})$ determined from Henry’s law [10,11]:

(30) $$H_{CO_{2-waer}}\;\left(\frac{mol}{m^{3}Pa}\right)=exp\left(-2044/T\right)/3.54\times 10^{-7}$$

The Henry’s constant for CO_2_ in aqueous PG is determined by

(31) $$H_{CO_{2}-PG}=H_{CO_{2}-water}\times 10^{\left(\alpha *C_{PG}\right)}$$

(32) $$\mathrm{Where}\;\alpha \;\left(\frac{m^{3}}{mol}\right)=\frac{62.183}{T}$$

The reversible reaction rate is considered first order with respect to rich PG ($C_{Pg-CO_{2},ts}$) in the tube side of the stripping unit

(33) $$r_{CO_{2}}=k_{r}\;C_{Pg-CO_{2},ts}$$

where $k_{r}$ is the reversible reaction rate constant.

#### 2.2.2. Membrane Layer

The transport of the regenerated solute gas (CO_2_) and the sweep gas (N_2_) components in the membrane section restrained between $r_{1}$ and $r_{2}$ can be designated by the material balance equation (Equation (34)), where diffusion is the only transport mechanism in the membrane phase [38]:

(34) $$\frac{\partial C_{CO_{2},ms}}{\partial t}=D_{CO_{2},ms}\left[\frac{\partial^{2}C_{CO_{2,ms}}}{\partial r^{2}}+\frac{1}{r}\frac{\partial C_{CO_{2},ms}}{\partial r}+\frac{\partial^{2}C_{CO_{2},ms}}{\partial z^{2}}\right]$$

Equation (35) describes the material balance for the sweep nitrogen gas in the membrane section.

(35) $$\frac{\partial C_{N_{2},ms}}{\partial t}=D_{N_{2},ms}\left[\frac{\partial^{2}C_{N_{2,ms}}}{\partial r^{2}}+\frac{1}{r}\frac{\partial C_{N_{2},ms}}{\partial r}+\frac{\partial^{2}C_{N_{2},ms}}{\partial z^{2}}\right]$$

Equations (36) to (39) are the boundary conditions of the membrane film ($i:CO_{2},\;N_{2}$)

(36) $$\mathrm{at}\;z=z_{1},\;\frac{\partial C_{i},ms\;}{\partial z}=0$$

(37) $$\mathrm{at}\;z=z_{2},\;\frac{\partial C_{i},ms\;}{\partial z}=0$$

(38) $$\mathrm{at}\;r=r_{1},\;D_{i,mr}\frac{\partial C_{i},ms\;}{\partial r}=D_{i,tr}\frac{\partial C_{i},ts\;}{\partial r}$$

(39) $$\mathrm{at}\;r=r_{2},\;C_{i,ms}=C_{i,ss}$$

#### 2.2.3. Shell of the Module

Equations (40) and (41) express the steady-state mass transport of CO_2_ and sweep N_2_ gas in the shell side, respectively:

(40) $$\frac{\partial C_{CO_{2},ss}}{\partial t}=D_{CO_{2},s}\left[\frac{\partial^{2}C_{CO_{2,ss}}}{\partial r^{2}}+\frac{1}{r}\frac{\partial C_{CO_{2},ss}}{\partial r}+\frac{\partial^{2}C_{CO_{2},ss}}{\partial z^{2}}\right]-v_{z,s}\left(\frac{\partial C_{CO_{2},ss}}{\partial z}\right)$$

(41) $$\frac{\partial C_{N_{2},ss}}{\partial t}=D_{N_{2},s}\left[\frac{\partial^{2C}N_{2},ss}{\partial r^{2}}+\frac{1}{r}\frac{\partial \;C_{N_{2},ss}\;\;}{\partial r}+\frac{\partial^{2}C_{N_{2},ss}}{\partial z^{2}}\right]-v_{z,s}\left(\frac{\partial C_{N_{2},ss}}{\partial z}\right)$$

The velocity profile in the shell side is described by Happel’s free surface [39].

The relevant boundary conditions are as follows:

(42) $$\mathrm{at}\;z=z_{1},\;C_{N_{2},ss}=C_{N_{2},0}\;\;(\mathrm{inlet}\;\mathrm{of}\;\mathrm{sweep}\;\mathrm{gas}\;\mathrm{concentration})$$

(43) $$\mathrm{at}\;z=z_{1},\;\frac{\partial^{2}C_{i},ss}{\partial z^{2}}=0\;(\mathrm{convective}\;\mathrm{flux})$$

(44) $$\mathrm{at}\;r=r_{2},\;D_{i,ts}\;\frac{\partial C_{i},ss\;}{\partial r}=D_{i,ms}\;\frac{\partial C_{i},ms\;}{\partial r}\;(\mathrm{diffusive}\;\mathrm{flux})$$

(45) $$\mathrm{at}\;r=r_{3},\;\frac{\partial C_{i},ss\;}{\partial r}=0\;(\mathrm{symmetry})$$

The model governing equations were solved simultaneously using COMSOL software version 5.5 (COMSOL Inc., Stockholm, Swedon). The software implies a finite element method to solve the model equations. Table 2 outlined the values used in the model predictions.

## 3. Results and Discussion

**Figure 3:**
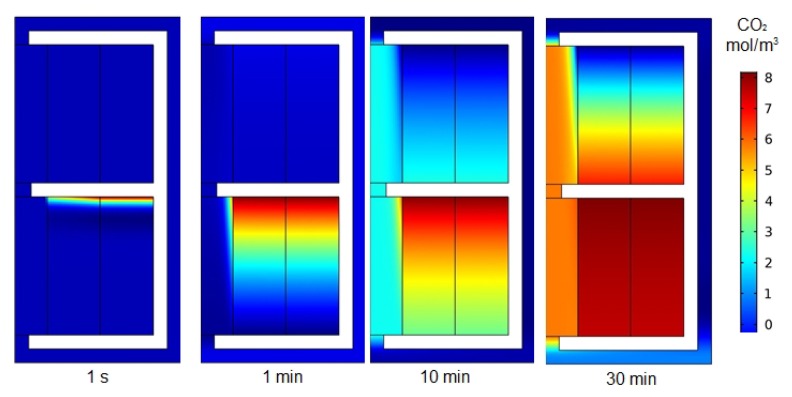
Surface plot for the CO_2_ concentration across the membrane module (absorber/stripper) with time. Liquid feed rate 150 mL/min, gas flow rate 20 mL/min, temperature 80 °C.

The surface plot of CO_2_ concentration across the membrane modules (absorption/stripping) is predicted in Figure 3. The inlet concentration of CO_2_ that enters the shell side of the absorption unit (bottom) is 8 mol/m^3^, and initial fresh solvent concentration (0.5 M PG) exists in the tube side. The CO_2_ being absorbed in the absorption unit with the PG solvent. The absorption efficiency declined with time. Initially, the CO_2_ concentration is the inlet of the membrane (absorber) shell side. With time (after 1 and 10 min), the percentage of CO_2_ absorbed dropped. The decreased in the absorption efficiency is due to the consumption of the PG solvent available for the absorption of CO_2_ in the hollow fiber lumen and the week regeneration of the rich PG in the stripping unit (top). After 30 min, almost no CO_2_ removal is observed in the absorber, the CO_2_ concentration across the absorber shell side is almost close to inlet concentration (8 mol/m^3^). This is attributed to the consumption of the available PG for the CO_2_ absorption and the week regeneration of the solvent. 

**Figure 4:**
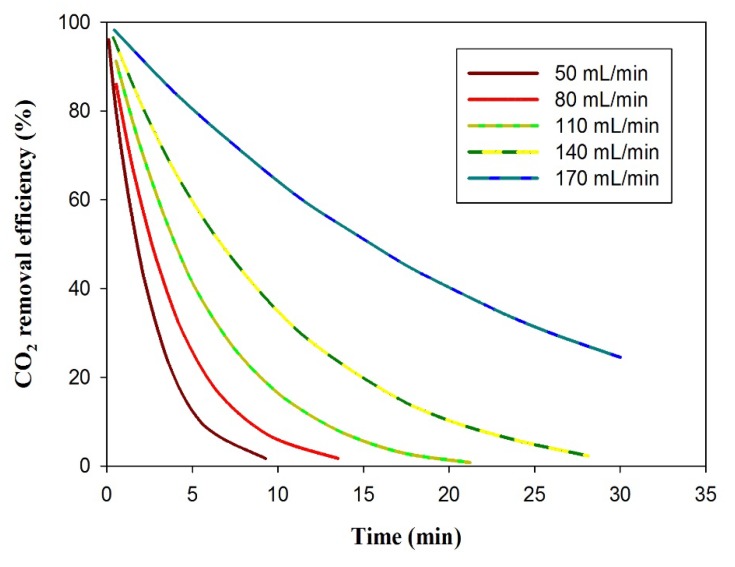
Effect of solvent initial feed rate (50, 80, 110, 140, and 70 mL/min) at constant gas feed rate (10 mL/min) on the CO_2_ removal efficiency with time.

Figure 4 describes the effect of the solvent flow rate in the absorption unit with time. At a low liquid circulation rate (50 mL/min), no noticeable percentages of CO_2_ removal were observed after 10 min of operation due to the consumption of PG accessible for CO_2_ absorption. As the fresh solvent circulation rate increased to 110 mL/min, the reachable solvent to remove more CO_2_ increased. After 30 min of operation, and a solvent circulation rate of 170 mL/min, 30% CO_2_ removal efficiency was reached. This phenomenon is expected because when the solvent circulation amount increases, there is sufficient fresh solvent in the membrane to capture more CO_2_ gas.

**Figure 5:**
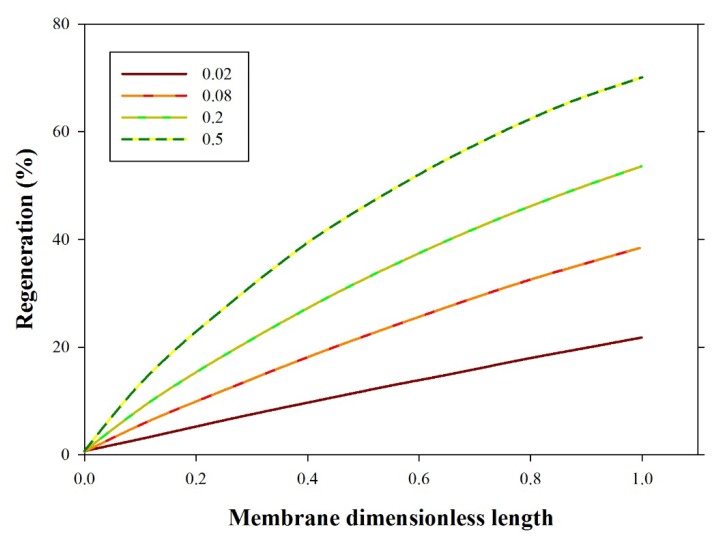
Stripping efficiency versus membrane dimensionless length at variable CO_2_ loading in the initial solvent (0.02, 0.08, 0.2, and 0.5 mole CO_2_/mole PG), liquid flow rate = 10 mL/min, gas flow rate = 100 mL/min).

The stripping efficiency as a function of CO_2_ loading in aqueous PG is demonstrated in Figure 5. The results revealed that the stripping efficiency increased with CO_2_ loading. Regeneration efficiency increased with increased initial CO_2_ loading. That is attributed to an increased CO_2_ concentration gradient. Based on Fick’s first law, the molar flux is directly proportional to the gradient of the CO_2_ concentration. Accordingly, the increase in the initial concentration of CO_2_ in rich solvent increases regeneration efficiency; simulation predictions agree with previously published work [43,44,45,46].

**Figure 6:**
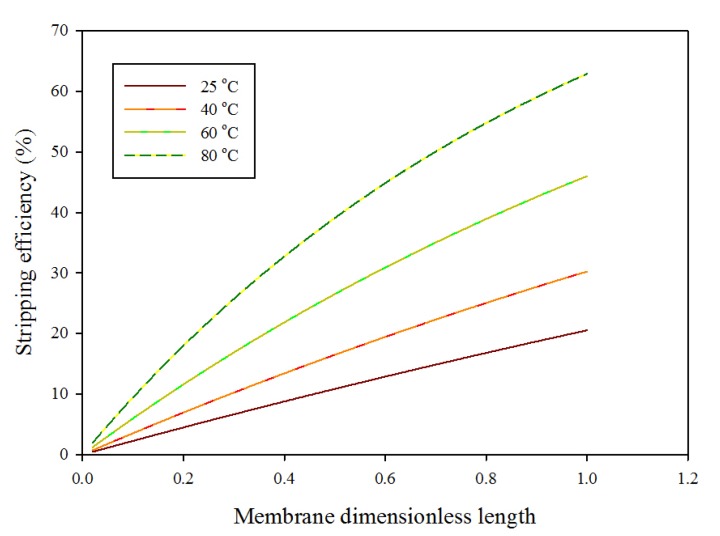
Stripping efficiency versus membrane dimensionless length at variable liquid solvent temperatures (25, 40, 60, and 80 °C); inlet gas flow rate is 600 mL/min and flow rate of inlet solvent is 20 mL/min.

The influence of rich solvent inlet temperature on regeneration efficiency along membrane dimensionless length is illustrated in Figure 6. As the stripping temperature of rich solvent increases, stripping efficiency increases. That is accredited to the decrease of CO_2_ solubility in rich solvent with temperature; CO_2_ solubility is inversely proportional to the temperature of solvent. At high temperatures, the dissolved CO_2_ in the rich solvent escapes and increases the stripping driving force of CO_2_ mass transfer, and more CO_2_ released leads to higher CO_2_ stripping efficiency [1]. 

**Figure 7:**
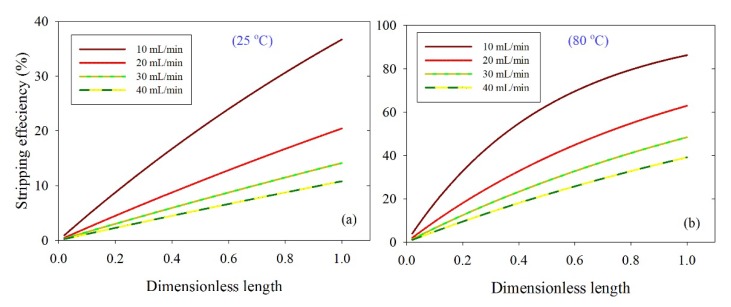
Effect of solvent circulation rate versus module dimensionless length on solvent stripping efficiency at gas flow rate 100 mL/min, and solvent temperatures 25 °C (**a**) and 80 °C (**b**).

The effect of solvent liquid flow rate on stripping efficiency along the membrane dimensionless length at 25 and 80 °C is depicted in Figure 7. Results revealed that the increase in solvent flow rate at low temperature (25 °C) has an insignificant increase in the CO_2_ stripping efficiency; by contrast, at high temperatures, there is a significant increase in the stripping efficiency. That is attributed to the noteworthy impact of temperature on the CO_2_ solubility of absorbed CO_2_ in liquid solvents. As previously mentioned, the CO_2_ solubility is inversely proportional to temperature. Hence at high temperatures, the CO_2_ solubility decreases and CO_2_ escapes from the solvent and is brushed by nitrogen-sweeping gas. 

**Figure 8:**
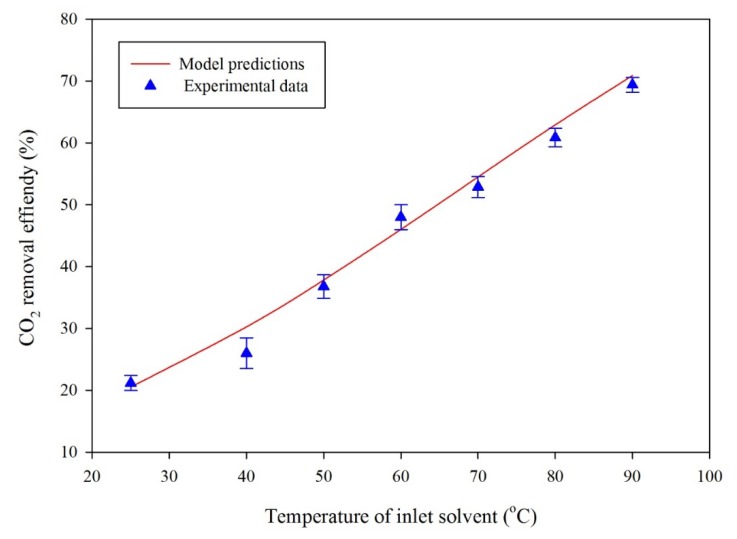
Effect of solvent temperature on CO_2_ removal efficiency. Hollow fibers were made of 28% PVDF/72% Triacetin, module (length is 260 mm, inner diameter is 8 mm), hollow fibers (ID/OD: 0.42/1.1 mm), sweep gas is nitrogen. Initial CO_2_ concentration in rich PG is 0.54 mol/L. Liquid flow rate is 20 mL/min and gas flow rate is 600 mL/min. The experimental uncertainty of CO_2_ percent removal is ±1.57.

**Figure 9:**
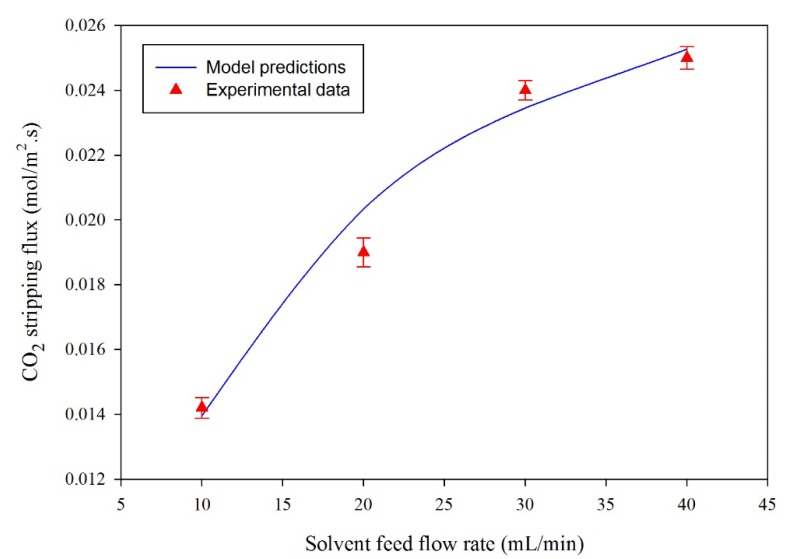
Effect of solvent feed rate on the flux and CO_2_ removal flux. Fiber made of 28% PVDF, 72% triacetin, module (length is 260 mm, inner diameter is 8 mm), hollow fibers (ID/OD: 0.42/1.1 mm). Initial CO_2_ concentration in rich PG is 0.54 mol/L. Solvent feed temperature = 80 °C and gas feed flow rate is 100 mL/min. The experimental uncertainty of CO_2_ removal flux is ±0.0005.

For the model validation, the experimental data of the CO_2_ percent removal efficiency as a function of temperature (Figure 8) and CO_2_ removal flux as a function of liquid feed flow rate (Figure 9) were plotted and compared with the simulation predictions. The experimental results are presented with standard error bars. Figure 8 illustrates the influence of rich solvent temperature on the percentage removal of CO_2_. The experimental results agreed with simulation predictions. As the PG rich solvent temperature increases, the removal percentage of CO_2_ increases due to decreasing CO_2_ solubility in liquid with temperature.

Figure 9 illustrates the comparison between experimental data and simulation results for the effect of the solvent circulation rate on CO_2_ stripping flux. The temperature and gas feed rate were kept constant at 80 °C and 100 mL/min, respectively. The experimental results were presented with standard error bars. Based on the experimental error bars in Figure 8 and Figure 9, the experimental results and simulation predictions were in good agreement between the experimental and simulation results. The percent efficiency was calculated as follows:

(46) $${\mathrm{CO}}_{2}\;\mathrm{removal}\;\mathrm{efficiency}\;(\%)=\left(\frac{\;C_{CO_{2,\;in}}-C_{CO_{2,\;out}}}{C_{CO_{2,in}}}\right)$$

The CO_2_ stripping flux is calculated as per Equation (47):

(47) $${\mathrm{CO}}_{2}\;\mathrm{removal}\;\mathrm{flux}=\left(\frac{\;\left(C_{CO_{2,\;in}}-C_{CO_{2,\;out}}\right)F_{g}}{A}\right)$$

where the $F_{g}$ is the inlet gas feed rate (m^3^/s), A is the total hollow fiber surface area

(48) $$A=2\pi r_{2}L*n$$

*L* is the length of the hollow fiber and *n* is the total number of fibers, $C_{CO_{2,in}}$ and $C_{CO_{2,out}}$ are the inlet and exit CO_2_ concentrations at experimental temperature and pressure in mol/m^3^. 

## 4. Conclusions

The absorption of CO_2_ in lean aqueous PG and the stripping of CO_2_ from rich PG were simultaneous modeled and solved using COMSOL Multiphysics version 5.5. The trend of the model predictions were in good agreement with experimental data. Results revealed that the stripping efficiency was enhanced with increased solvent temperature, solvent circulation rate, and CO_2_ initial concentration in rich solvent. The effect of the solvent circulation rate on stripping efficiency at low temperature was insignificant; by contrast, there is ra emarkable increase in the stripping efficiency with the solvent circulation rate at high solvent temperature. A high solvent circulation rate increases the efficient stripping time of the absorption/stripping solvent–gas membrane contactor module. 

## Figures and Tables

**Table 1:** Dimensions membrane module [1].

Property	Value
Inner hollow fiber diameter (mm)	0.42
Outer hollow fiber diameter (mm)	1.10
Number of fibers	15
Inner surface area (m^2^)	$5.15\times 10^{-3}$
Outer diameter of module (mm)	8.0
Effective length module (mm)	260

**Table 2:** Parameters of the process.

Parameters	Value	Ref.
Reversible reaction rate constant, $k_{r}$ (1/s)	3.4 × 10^3^exp(−2800/T)	calculated
Diffusivity of CO_2_ in shell side, $D_{CO_{2},s}$ (m^2^/s)	8.3 × 10^−10^ × T^1.75^	[40]
Diffusion of CO_2_ in tube side, $D_{CO_{2},t}$ (m^2^/s)	1.5 × 10^−6^ × exp(−2119/T)	[36]
Diffusivity of CO_2_ in membrane, $D_{CO_{2},m}$	$D_{CO_{2},t}$ × ε/τ	[41]
Porosity, *ε*	0.4	Measured
Tortuosity, *τ*	(2 − ε)/ε	[42]
